# Association among activities of daily living, instrumental activities of daily living and health-related quality of life in elderly Yi ethnic minority

**DOI:** 10.1186/s12877-017-0455-y

**Published:** 2017-03-22

**Authors:** Lingyun Ran, Xiaodong Jiang, Baogang Li, Hongqian Kong, Mengqi Du, Xiaolan Wang, Hua Yu, Qin Liu

**Affiliations:** 1 Nursing School of Kunming Medical University, Kunming, Yunnan Province China; 2 Chongqing Three Gorges Medical College, Chongqing, China; 3 Public Health School of Kunming Medical University, Kunming, China; 4 No.1 High School, Kunming, Yunnan China; 5 Tumor Hospital of Yunnan Province, The Third Affiliated Hospital of Kunming Medical University, Kunming, Yunnan China

**Keywords:** Health-related quality of life, Elderly, Yi ethnic minority, ADL, IADL

## Abstract

**Background:**

The health-related quality of life (HRQoL) of the elderly population of Yi ethnic minority, which is the seventh largest nationality in China, has been rarely reported. This study was designed to explore the HRQoL of the elderly Yi ethnicity and association between their HRQOL and functional abilities.

**Methods:**

A total of 291 Yi ethnic residents were randomly recruited from 12 rural counties in Yunnan province and divided into different age groups. Local residents in Yunnan province and the elderly from Hangzhou were enrolled as controls. The MOS 36-Item Short Form Health Survey (SF-36), activities of daily living (ADL), instrumental activities of daily living (IADL) scales were utilized to evaluate the HRQoL and functional ability. One-way ANOVA was used to statistically compare the ADL and IADL among different age groups. The influential variables on HRQOL were analyzed by multiple linear regression analysis. Pearson correlation analysis was used to analyze the association among HRQoL, ADL and IADL.

**Results:**

The HRQoL of the elderly Yi minority was significantly lower than those of local residents in Yunnan province and the elderly counterparts in Hangzhou. The IADL ability of the elderly Yi minority was low, whereas they could perform most items of ADL. ADL, IADL, and education level were positively associated with HRQoL, whereas age, chronic diseases, and the frequency of medication use were negatively correlated with HRQoL.

**Conclusion:**

The HRQoL and functional capacity of the elderly Yi ethnic minority were lower compared with their counterparts in Yunnan province and Hangzhou. The low level of IADL indicated that the elderly Yi participants had a high risk of cognitive impairment. Much attention should be diverted to influential factors of the HRQoL.

**Electronic supplementary material:**

The online version of this article (doi:10.1186/s12877-017-0455-y) contains supplementary material, which is available to authorized users.

## Background

Globally, the aging population in developing countries is increasing at twice the pace of that in developed nations. As the most populous country worldwide, China is encountering the massive challenge of aging population. The statistics data of Chinese Sixth National Census demonstrated that the elderly population (aged 65 and above) in 2010 was 118.83 million, accounting for 8.87% of the total population. The percentage of people aged ≥65 years was increased by 1.91% compared with that of the Fifth National Census in 2000 [1]. It has been estimated that the aging population will be 324 million by 2040 in China, accounting for 22.56% of the total population [2].

As the seventh most populous nationality in China, the population of Yi ethnic minority is approximately 8.71 million among 56 different ethnics in China. Most of the Yi ethnic minority resides in the southwestern parts of China including Yunnan, Sichuan, and Guizhou provinces. Approximately 57.59% of the Yi minority lives in different parts of Yunnan province and acts as the most populous ethnicity in Yunnan province. The distribution of age of the Yi ethnicity is as follows: 30.27% for 0–14 years old, 64.94% for 15–64 years old, and 4.79% for the elderly aged 65 or above. The mean life expectancy of the Yi ethnicity is estimated to be 63.96 years [3].

The elderly population is likely to suffer from health problems and irreversible decline in functional abilities [4, 5]. One of the essential issues of the senior adults is the health-related quality of life (HRQoL) that includes physical and mental health perceptions and their correlation with health risks and conditions, functional status, social support and socioeconomic status [6]. HRQoL is associated with both self-reported chronic diseases, such as diabetes mellitus, breast cancer, arthritis, and hypertension, and their risk factors including body mass index, physical inactivity, and smoking status, *etc.* [7]. The burden of preventable diseases, injuries, and disabilities can be determined by measuring HRQoL and the relationship between HRQoL and risk factors can be estimated with valuable new insights by the assessment of HRQoL. Multiple measures have been adopted to assess HRQoL and related concepts of functional status. Among them are the Medical Outcomes Study Short Forms (SF-12 and SF-36), the Sickness Impact Profile, and the Quality of Well-Being Scale, World Health Organization Quality of Life BREF (WHOQOL-BREF) [8, 9].

Until date, a few studies have been conducted to analyze the influential factors of HRQoL in the elderly population. Luthy et al. reported that male gender was associated with better HRQoL, whereas their female counterparts were manifested with specific problems with aging, such as loneliness, handicaps, a longer duration of symptoms prior to death. They also revealed that higher educational background was associated with better HRQoL [10]. Varma et al. demonstrated that the elderly Indians living at their homes yielded higher HRQoL compared with those residing in the community [11]. Kharicha et al. revealed that the aged population living alone was more prone to obtain fair or poor HRQoL than those who resided with family members in a cross-sectional study [12]. A 9-year prospective study in Japan revealed that both physical and mental health conditions were influenced by transitions in living arrangement [13]. Moreover, the contact with family members and friends and the extent of social activities were significantly negatively with mental health decline among women living alone in America [14].

Chinese researchers have analyzed the HRQoL of the elderly population living in different regions in recent years. Wu et al. reported that health status and HRQoL of the senior individuals in Bazhong were significantly poorer than that of the rural elderly in the same province after the flood disaster [15]. Another study focusing upon the association between living arrangement and HRQoL of the elderly residents from urban areas revealed that the elderly residents living with a spouse alone obtained better HRQoL. Moreover, social interaction was positively correlated with the score of HRQoL [16]. Liang and Wu identified that the male senior adults obtained better HRQoL compared with their female counterparts, and the senior residents with higher educational level had better HRQoL [17]. Some researchers already reported that need for help with daily living, multiple numbers of diseases, and loneliness are associated with lower HRQoL [18, 19]. However, the association between functional abilities and HRQoL in the elderly minor ethnicity has not been investigated. This study aims to assess the ADLs and evaluate the self-rated HRQoL of the senior Yi ethnicity, and investigate the influential factors related to the HRQoL.

## Methods

### Sampling collection

In total, 291 Yi ethnicities, aged 60 years old or above, from 12 rural counties from Yunnan province were recruited in this study using a multi-stage random sampling method. Inclusion criteria were as follow: Yi elderly; age 60 and above; the mental ability to answer the interview questionnaire. The Yi elderly individuals suffered from mental disorders or communication barriers would be excluded from this study. A total of 291 elderly Yi ethnicities responded (response rate 100%) and were face-to-face interviewed by trained researchers from Kunming Medical University of Yunnan province who were also the Yi ethnicity but could speak both Mandarin and Yi language. Since the Yi elderly individuals could not speak or understand Mandarin, they had to answer the questions read by the interviewers who were also Yi ethnicity using Yi language. The interviews were a series of questions and scales, for example, age, number of chronic diseases, categories of medication, education level, activities of daily living (ADL), instrumental activities of daily living (IADL), and health-related quality of life (HRQoL).

### Ethical considerations

This research was approved by the local government permission from different Yi villages. Written and verbal informed consents were obtained from all participants who were volunteering prior to this survey. All personal information of the participants was assured to be kept confidentially.

### Measurement instruments

ADLs: the functional capacity of the elderly can be measured by Activities of Daily Living (ADL) scale that is widely used in clinical studies and community-based researches [20]. Two ADL tools were applied into present study. One is the Barthel Index, a widely used ADL tool to assess basic activities of daily living of the elderly, which measures functional levels of self-care and mobility and rates the ability to feed and groom oneself, bathe, go to the toilet, walk or use a wheelchair, climb stairs, and control bowel and bladder. The score of ADL varies from 0 to 100 in accordance with older adult’s ability to do the daily activities on his or her own. A result of 0 stands for complete dependency in activities of daily living; whereas the result of 100 means complete independent in activities of daily living [21]. Another is Instrumental Activities of Daily Living (IADLs) scale, which include a range of activities that are considered to be more complex with ADLs and address the older adult’s ability to interact with his or her environment and community. IADLs include the ability to use the telephone, cook, shop, do laundry, housekeeping, manage finances, take medications, and prepare meals. The scores of this scale ranged from 0 to 8, with 0 being the least independent and 8 being the most independent in the above eight items [22]. The internal consistency was measured by Cronbach α coefficient, which was 0.739 for both of these two ADL scales (Additional file 1).

HRQoL: as above indicated, SF-36 scale is a reliable tool to assess the health-related quality of life in different population. SF-36 contains eight health concepts that are distributed in 36 items. The concepts are as follow: physical functioning (PF), role limitations due to physical problems (RP), bodily pain (BP), general health (GH), vitality (VT), social functioning (SF), role limitations due to emotional problems (RE), and mental health (MH). The Physical Component Summary includes PF, RP, BP, GH and Mental Component Summary contains VT, SF, RE, and MH. The scores of each domain range from 0 to 100, with a higher score standing for better HRQoL [23]. The result of internal consistency reliability SF-36 was 0.842 by calculating the value of Cronbach α coefficient.

### Other variables

Other variables in this survey included the self-designed social-demographics questions, such as age, gender and educational background. The Yi ethnic elders were divided into five age groups (60–64, 65–69, 70–74, 75–79, 80 years and older) and educational background was classified into four categories that included illiterate, primary school, junior high school, senior high school and above. We also examined the history of chronic illnesses and the number of medication categories in this population. Yes or no choices were given to the participants to answer whether they had chronic diseases at the research period or not. There were four categories of the number of medication types, including less than three, three to five, six to ten, and ten and above.

### Statistical analysis

Data base was established by Epidata 3.1 and statistical analysis was performed using the Statistical Package for Social Sciences (SPSS 17.0, SPSS Inc., Chicago, USA). Descriptive statistics such as frequencies, means and standard deviations were first obtained to explore the demographic characteristics of chronic diseases. ANOVA was used to examine the statistical differences of variables among different age groups. Pearson correlation analysis and multiple linear regression analysis were utilized to assess the effect of ADL, IADL, and other social-demographic variables on SF-36, by utilizing total score of SF-36 as a dependent variable. A *P* value of less than 0.05 was considered as statistical significance.

## Results

### Baseline demographic and clinical characteristics

Among a total of 291 subjects, there were 110 females (37.8%) and 181 males (62.2%), and the mean age of this population was (69.0 ± 6.2) years old. Among them, 56.7% of the enrolled participants received primary education. Approximately 60.1% of them had a medical history of chronic diseases. In addition, 58.4% of the participants received less than 3 categories of medications. The social-demographic as well as clinical characteristics of the study population are illustrated in Table 1.

**Table 1:** Socio-demographic and clinical characteristics of the enrolled population (*n* = 291)

	Number (percent, %)
Gender	
Male	181 (62.2)
Female	110 (37.8)
Age-group	
60–64	77 (26.5)
65–69	85 (29.2)
70–74	65 (22.3)
75–79	45 (15.5)
80 years and above	19 (6.5)
Education level	
Illiterate	78 (26.8)
Primary school	165 (56.7)
Middle school	23 (7.9)
High school	25 (8.6)
Chronic diseases	
Yes	175 (60.1)
No	116 (39.9)
Categories of medication	
<3	170 (58.4)
3 to 5	102 (35.1)
6 to 10	16 (5.5)
>10	3 (1.0)

### Comparison of ADL and IADL scores among different age groups

The scores of ADL and IADL among different age groups are demonstrated in Table 2. The average ADL score in this population was calculated as 92.2 ± 16.1, suggesting that the elderly Yi ethnicity could perform main daily activities independently. The percentage of the Yi elderly who were independent in ADL was calculated as 95.1% in the 65–69 years age group, and 87.6% in the >80 years group. Notably, the ADL score in the 65–69 years age group was the highest among all groups. The ADL score in the 75–79 years group was higher compared with that of the 70–74 years group. The mean ADL score of the elderly Yi participants was 4.7 ± 1.8, suggesting that they could not fulfill instrumental daily activities independently. The average IADL score was 5.2 in the 60–64 years group, significantly descended to 3.4 in the >80 years group, except the IADL score in the 75–79 age group. The mean ADL and IADL scores significantly differed among different age groups (all *P* < 0.05).

**Table 2:** One-way ANOVA of ADL and IADL scored among different age-groups

Age-group (year)	ADL(mean ± SD)	IADL(mean ± SD)
60–64	94.4 ± 12.3^a^	5.2 ± 1.5^a^
65–69	95.1 ± 10.0	5.0 ± 1.8
70–74	87.5 ± 21.4	4.2 ± 1.7
75–79	92.0 ± 19.8	4.8 ± 2.0
80 and above	87.6 ± 17.4	3.4 ± 1.8
Total	92.2 ± 16.1	4.7 ± 1.8
*P* value	0.02	0.000

^a^denotes statistical significance compared with other age groups

### Comparison of eight domains and total scores of SF-36 scale among different age groups

Eight domains and total scores of SF-36 among all age groups were revealed in Table 3. The mean total score of SF-36 was 46.8, and the scores of physical and mental health were 47.8 and 45.8 in this Yi elderly sample. The highest score was SF (62.6 ± 15.8), followed by PF (57.5 ± 23.2), MH (57.2 ± 13.4), VT (55.8 ± 12.7), BP (46.3 ± 13.5), GH (46.1 ± 14.8), RE (42.0 ± 41.1), and RP (35.9 ± 37.8). These results indicated that the HRQoL was not favorable in this population.

**Table 3:** Descriptive data of eight domains and total score of SF-36 scale among different age groups

Domain	60–64 year group	65–69 year group	70–74 year group	75–79 year group	80–84 year group	*P* value	Total sample
PF	68.0 ± 23.3	56.1 ± 20.3	55.2 ± 22.4	54.4 ± 21.5	36.6 ± 24.2^a^	0.000	57.5 ± 23.2
RP	45.8 ± 36.8	32.4 ± 36.8	32.3 ± 36.8	33.3 ± 40.2	30.3 ± 43.8	0.124	35.9 ± 37.8
BP	46.9 ± 16.3	44.7 ± 10.2	46.4 ± 11.5	46.7 ± 13.8	49.3 ± 13.8	0.683	46.3 ± 13.5
GH	50.2 ± 16.5	44.6 ± 14.9	44.0 ± 12.3	46.0 ± 12.6	43.7 ± 17.9	0.078	46.1 ± 14.8
VT	57.7 ± 15.1	54.3 ± 10.1	54.4 ± 10.8	59.3 ± 12.5	51.3 ± 17.0	0.051	55.8 ± 12.7
SF	61.4 ± 15.7	62.8 ± 13.7	63.8 ± 14.8	63.3 ± 17.4	61.2 ± 23.9	0.890	62.6 ± 15.8
RE	49.8 ± 40.3	38.8 ± 40.4	41.5 ± 40.4	40.7 ± 44.3	29.8 ± 41.4	0.291	42.0 ± 41.1
MH	56.4 ± 14.2	56.4 ± 13.4	56.7 ± 12.0	60.6 ± 13.9	58.3 ± 13.3	0.442	57.2 ± 13.4
Physical health	51.2 ± 8.2	46.9 ± 7.0	46.8 ± 6.4	47.1 ± 7.4	43.2 ± 8.5^a^	0.000	47.8 ± 7.7
Mental health	46.0 ± 6.5	45.2 ± 5.1	45.4 ± 5.1	47.3 ± 6.7	44.6 ± 6.2	0.265	45.8 ± 5.8
Total score of SF-36 scale	48.6 ± 6.6	46.0 ± 5.2	46.1 ± 4.5	47.2 ± 6.0	43.9 ± 6.6^a^	0.005	46.8 ± 5.8

^a^denotes statistical significance compared with other age groups

### Comparison of HRQoL among the elderly Yi, Yunnan local residents and the elderly in Hangzhou

**Fig 1:**
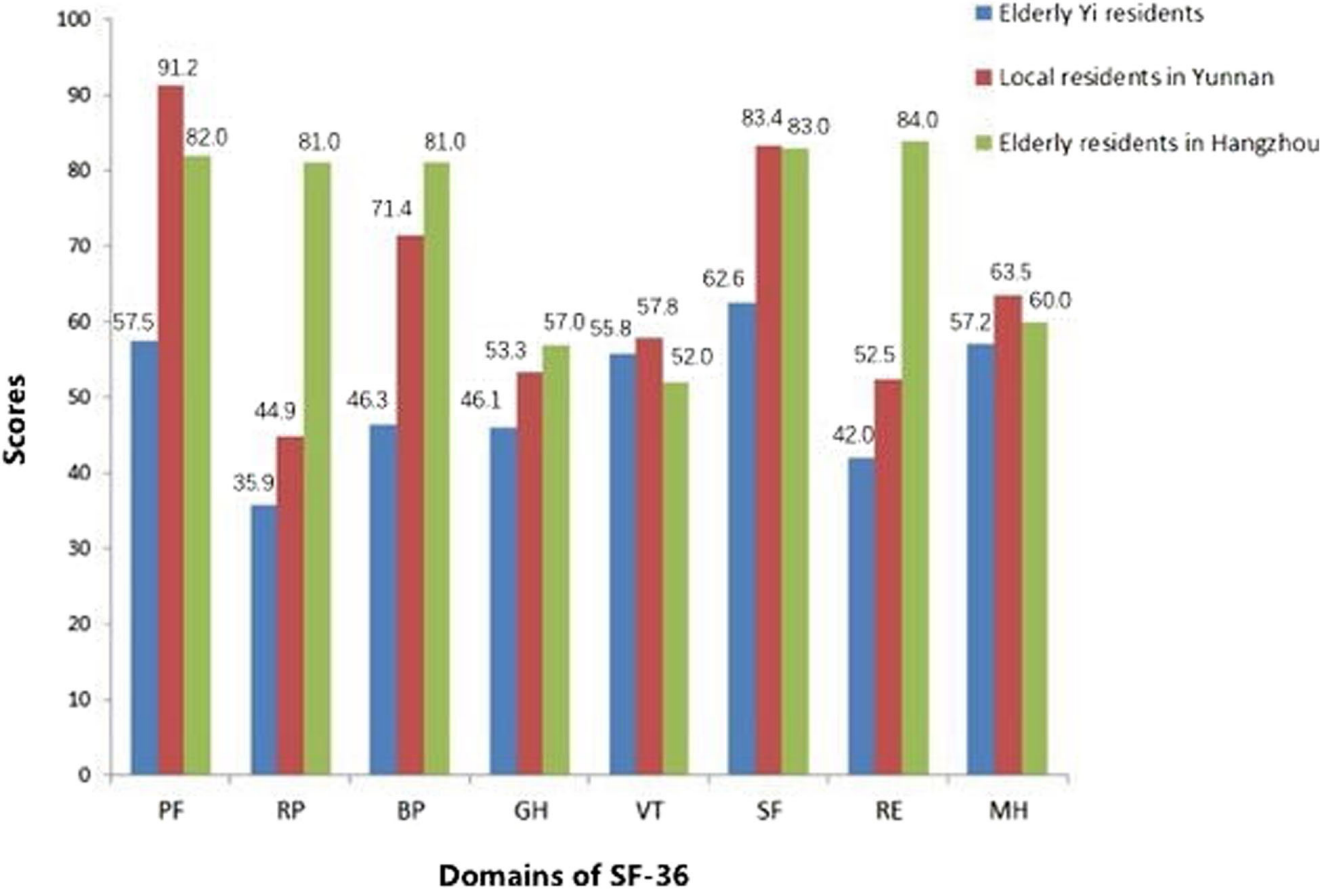
Comparison of HRQoL among the elderly Yi, Yunnan local residents and the elderly in Hangzhou

The mean scores of eight domains of SF-36 among Yi elderly, Yunnan local residents, and the elderly in Hangzhou were statistically compared in Fig. 1. The norms of Yunnan local residents and the elderly in Hangzhou served as the references since there was no normative valve of SF-36 in the elderly residents from Yunnan province. The HRQoL of the Yi elderly was significantly lower than that of Yunnan local and the aged residents in Hangzhou, as illustrated in Fig. 1. P values in the first seven domains, that are PF, RP, BP, GH, VT, SF, RE, are less than 0.01. *P* = 0.05 in MH domain.

### Multiple linear regression analysis of influential variables on HRQoL

Multiple linear regression analysis evaluated the influential factors related to HRQoL, as illustrated in Table 4. The findings demonstrated that high education level was related to a good HRQoL (*P* < 0.05), suggesting that higher educational background was associated with better HRQoL. Nevertheless, the other three variables were negatively correlated with the HRQoL in this group including the incidence of chronic diseases, age, and category of medication (*P* < 0.05). The results implied that the incidence of chronic diseases, advanced age, and more types of medication contributed to a worse HRQoL.

**Table 4:** Multiple linear regression analysis of influential variables on HRQoL (*n* = 291)

	Unstandardized regression coefficient	Robust standard errors	Standardized regression coefficient	t value	*P* value
Constant	68.952	4.899	-	14.075	0.000
Chronic diseases	−2.226	0.942	-.143	−2.363	0.019
Age	-.289	0.070	-.237	−4.145	0.000
Education level	1.590	0.527	0.169	3.017	0.003
Category of medication	−1.992	0.703	-.170	−2.834	0.005

*R* ^*2*^ = 0.75

### Correlation analysis among HRQoL and ADL, IADL

Pearson correlation analysis was utilized to analyze the association among HRQoL, ADL and IADL, respectively. The results showed that both ADL and IADL were positively correlated with HRQoL (both *P* < 0.05), indicating that the more independent level in the ADL and IADL, the higher level the HRQoL was.

## Discussion

To our knowledge, this is the first research to evaluate the HRQoL of ethnic elderly in China. The results indicated that the HRQoL of Yi ethnic elders was significantly worse compared with that of the Yunnan local residents and the elderly in developed areas of China, which is consistent with other researches in China and other countries [11, 24]. IADL problems, low education level, and outdated economy, culture, and healthcare contributed to the low HRQoL in this population. Education level, ADL, IADL scores were positively associated with better HRQoL. However, chronic diseases, older age, and category of medication were negatively related to worse HRQoL.

It is widely accepted that ADL ability including basic ADL and IADL decreases with age. However, significant difference exists among different age groups. Our findings on Yi ethnic elderly identified that the ADL ability in the 75 years age-group did not abide by the age rule, which is consistent with the research in Japan, which reported that 80 years old group were different in many items of ADL [25]. The ADL and IADL are positively associated with HRQoL. Bryła et al. found that the independent city-dwelling elderly obtained twice the HRQoL than that of the disabled elders in the same situation [26]. Our study also yielded the same observation. Moreover, our study confirmed that aged residents with good functioning in ADL showed a great dependency upon IADL. The research by Rensbergen and Pacolet identified this issue in their study that the older subjects obtained significantly lower scores in IADL though they scored higher in basic ADL [27]. The current study implies that these Yi participants might have a high risk of cognitive impairment since the poor functioning in IADL. Nygard reported that IADL can be impaired before the beginning of dementia and Jekel et al. also found that deficits in IADL were present congruently in mild cognitive impairment [28, 29]. Another study also discovered that the greater the IADL need is, the sooner people move into an institution. Therefore, some researchers suggested that supporting the IADL needs is the best guarantee to delay or prevent institutionalization [30].

Aged people are influenced by social, biological, mental, economic, and medical problems so the HRQoL of the elderly depends on many factors [26]. Many previous studies confirmed that better education was connected with more healthy life. The research of quality of life of residents in 10 nursing homes by Tseng and Wang concluded that quality of life relies on education [30]. Bryła et al. found that the elderly with a university education got twice the score for quality of life than those without a university education [26]. Our results also witnessed the impact of education on HRQoL. The majority (56.7%) of our samples only received elementary education, and 26.5% of them were illiterate so these could explain poor HRQoL among them. Age and chronic diseases are also factors can decline the quality of life. Our findings revealed that older age, chronic diseases, and more number of medications lead to poor HRQoL. Wu et al. reported that poor quality of life and health were related to older age, chronic diseases, being single and/or poor sleep patterns, *etc* [15].

Yunnan is the one of the least economically and culturally developed provinces in China. In total, twenty six ethnic groups inhabit in Yunnan province including the Yi ethnicity. As a matter of fact, most of the ethnic groups live in the more outdated areas in Yunnan province than the Han people, as the major nationality in China. In 2010, the life expectancy of people from the Yi ethnic group was 63.96 years, significantly shorter compared with 74.8 years of Chinese people from other ethnicities across China [31]. HRQoL of the aging population also depends on economic support, social services, and healthcare services. Some researchers identified that social help positively affected person’s quality of life, while the absence of financial support, social help and medical services negatively connected with people’s HRQoL [32, 33]. Actually, the above items of Yunnan province lag far behind those of the developed areas in China. Consequently, the HRQoL and life expectancy of the Yi elderly were significantly worse compared with their counterparts living in developed areas. Concerning of the implication on the policy and nursing service, the Yi elderly in rural counties should be provided with timely social assistance to offer them more independence and improved HRQoL in all areas [34, 35].

This study had several limitations. First, the Yi elderly were from the same province which may not generalize the whole population. Second, the sample size was not large enough (*n* = 291). Future studies will focus on these limitations.

## Conclusion

Taken together, the HRQoL and functional capacity of the elderly Yi ethnic group were worse compared with those of the local residents in Yunnan province and the elderly living in Hangzhou, an economically developed city in China. The subnormal level of IADL may indicate the elderly Yi ethnic population suffers from a high risk of cognitive impairment. Financial, social and medical services should be delivered to resolve the issues of their age, educational background, chronic diseases, ADL and IADL assistance.

## Additional file

Additional file 1: Table S1. Items and description in the ADL Scale. **Table S2.** Items and description in the IADL Scale. (DOC 37 kb)

## Abbreviations

ADL: Activities of daily living
HRQoL: Health-related quality of life
IADL: Instrumental activities of daily living
WHOQOL-BREF: World Health Organization Quality of Life BREF
