# Supplementary material for: Association among activities of daily living, instrumental activities of daily living and health-related quality of life in elderly Yi ethnic minority
Source: BMC Geriatr. 2017 Mar 22;17:74. doi: 10.1186/s12877-017-0455-y (PMC5361829; doi:10.1186/s12877-017-0455-y)
Supplement: Additional file 1: Table S1. — Items and description in the ADL Scale. Table S2. Items and description in the IADL Scale. (DOC 37 kb) [file 12877_2017_455_MOESM1_ESM.doc]

## Table S1 Items and description in the ADL Scale

| **Item** | **Description** |
| --- | --- |
| **Feeding** | Relates to how a resident eats and drinks, including other means of nourishment intake, such as tube feeding. |
| **Bathing** | Relates to how a resident takes a shower or tub bath, or sponge bath |
| **Grooming** | Includes how a resident keeps himself/herself clean and make his/her face, hair and skin look nice |
| **Dressing** | Includes how a resident puts on, fastens and takes off all items of street clothing. |
| **Bowels** | Relates how a resident control his/her bowel movement, including going to the toile to pass the bowel |
| **Bladder** | Relates how a resident control his/her urine, including going to the toile to pass the urine |
| **Transfer** | Includes how a resident moves between surfaces such as bed and chair. |
| **Toilet use** | This includes how a resident uses a toilet, commode, bedpan or urinal and transfer on and off a toilet. |
| **Mobility** | Includes how a resident moves between locations in their room and the corridor outside their room. |
| **Stairs** | Relates to how a resident climb or walk down the stairs |

## Table S2 Items and description in the IADL Scale

| **Item** | **Description** |
| --- | --- |
| **Ability to use telephone** | This relates to how a resident operates a telephone, looks up and dial numbers, answer telephone |
| **Shopping** | Relates to how a resident goes to shops or grocery stores or shopping malls to buy things |
| **Food preparation** | Includes how a resident plan, prepare and serve meals |
| **House keeping** | Relates to how a resident does the housework, e.g. wash dishes, make beds, mop floor, clean windows, etc. |
| Laundry | Includes how a resident uses the washing machine or wash the clothes by hands |
| Mode of Transportation | Includes a resident travels on public transportation or taxi or automobile |
| Responsibility for own medications | Includes a resident prepare and take medication |
| Ability to Handle Finances | Includes a resident makes the budget, writes checks, pays rent, pay bills, goes to bank, or keeps track of income |
